# Thermal stress-related physiological, behavioral, and serum biochemical responses in indigenous pigs adapted to Eastern Himalayan region

**DOI:** 10.3389/fvets.2022.1034635

**Published:** 2022-12-15

**Authors:** Kadirvel Govindasamy, Chamniugongliu Gonmei, Ningthoujam Suraj Singh, Nakambam Manoranjan Singh

**Affiliations:** Division of Animals and Fisheries Sciences, ICAR Research Complex for NEH Region, Umiam, Shillong, Meghalaya, India

**Keywords:** adaptation, behavior, indigenous, pig, thermal stress, response

## Abstract

**Introduction:**

The current study was carried out to investigate the effect of micro-environmental variations on physiological, behavioral, and serum biochemical parameters of indigenous (Niang Megha), Hampshire, and crossbred (75% Hampshire X 25% Niang Megha).

**Methods:**

Rectal temperature (T_R_), skin surface temperature (T_SS_), respiration rate (RR), and heart rate (HR) were recorded at 0,900 and 1,600 h weekly once for 2 months for each season in grower pigs of each genotype. CCTV video cameras were utilized to observe the behavioral changes. Five milliliters of blood samples was collected to estimate different biochemical parameters.

**Results:**

Season affected (*p* < 0.05) all physiological parameters which generally increased during summer except T_R_ and RR of indigenous pig. T_R_, T_SS_, RR, and HR were significantly (*p* < 0.05) higher for Hampshire than for indigenous and crossbred in the summer season. The frequency and behavioral activities to heat loss or to conserve heat such as shivering and wallowing were lower except for physical activity that was higher at all times in indigenous pigs. Seasonal variations influenced metabolic activity and serum activity of alkaline phosphatase (ALP) and alanine transaminase (ALT), which rose in summer in all genotypes. Serum ALP and thyroxine (T_4_) were significantly (*p* < 0.05) higher for indigenous pig in both the seasons. The insulin level was significantly (*p* < 0.05) higher in indigenous pigs with no significant difference between Hampshire and crossbred in summer whereas there was significant difference among the genotypes in winter. However, superoxide dismutase (SOD) showed no significant difference in the study. Indigenous pigs had the lowest serum cortisol concentrations, whereas Hampshire had the highest.

**Conclusion:**

The current study's findings on several parameters of three different genotypes suggest that indigenous pigs in this region are more adaptable to the region's changing climatic conditions.

## Introduction

The Eastern Himalayas are one of the most biologically rich areas on the Earth. In India, the Northeastern Hill (NEH) region represents the Eastern Himalayan region and has a total geographical area covering ~2,62,379 sq. km. area and lies between 21°34′-29°50′ N latitudes and 87°32′-97°52′ E longitudes in the Eastern Himalayan hill region (1). This region is well-known for the diverse culture of human races and home to a large number of ethnic people (2), providing a diverse range of habitats to about 225 tribes in India, out of 450 in the country (3). Agriculture is the primary source of income for the majority of the rural population of this region (85%), who conducts mixed farming, with livestock accounting for 18% of the value of output from the agricultural sector (4). Moreover, the livestock sector plays a major role in the socioeconomic development in this region, and it is the major source of livelihood for 20 million people, especially women (5), also an important contributor to sustainable food security for many nations, particularly in low-income areas and marginal habitats that are unsuitable for crop production (6). Among all the livestock, pig husbandry is one of the popular livestock and total pig population is mainly dominated by local non-descript pigs (65–75%) in the region (7). Indigenous pig breeds bear unique features such as better heat tolerance, disease resistance, good maternal qualities, early sexual maturity (8), and good quality bristles (9) compared with exotic and crossbreds. The body coat color of indigenous pigs of this region is predominantly black in color and has long and dense hairs extending from wither to the hindquarter.

Climate change is a major worldwide challenge and leads to various environmental stresses that have an impact on the production of livestock (10). Animals can adapt to climatic stressors by different types of adaptive mechanisms including genetic or biological adaptation, phenotypic or physiological adaptation, acclimatization, and habituation (11). However, the response mechanisms that ensure survival are detrimental to performance and productivity (12). The physiological changes when exposed to environmental stress can be measured by the variation in respiration rate, rectal temperature, heart rate, and skin surface temperature (13). Increased respiration or panting increases airflow and evaporation of water from the lungs and hence releases additional heat (14). The physiological response of the animal to its internal and external environment is also reflected in the blood profiles. Changes in hemato-biochemical parameters are useful methods for determining the degree of stress caused by environmental and dietary factors (15) and adaptability to given environmental conditions of an animal (16). Metabolic hormones affecting thermogenesis can also be estimated as the physiological index of environmental adaptation of an animal (17). Similarly, pigs exhibit natural behavioral responses to thermal stress, which include huddling together and shivering when cold, seeking shade, wallowing in water, and changing from diurnal to nocturnal feeding times when heat stressed during the day (18). Pigs have a thick subcutaneous adipose tissue layer and fewer sweat glands; therefore, pigs control their body temperature by behavioral thermoregulation instead of sweating (19). To enhance heat dissipation, they increase direct contact with cool surfaces (20), modifying their lying position, increased excretion, and wallowing in their excreta (21). According to anecdotal evidence, indigenous pigs of this region are better adapted to harsh climatic conditions and subsistence farming, but there is little scientific evidence to back up the statement. Indigenous breeds are more thermotolerant than crossbred and purebred animals in terms of the adaptation potential of the livestock species (22). Indigenous breeds that have evolved in tropical and subtropical regions have a higher adaptive capacity to such stress than exotic breeds. The South African indigenous Windsnyer pigs had better thermoregulatory mechanisms than the large white pigs of the temperate region in the semiarid climate of South Africa (23). Indian breeds of cattle such as *Bos indicus* perform well as compared to exotic cattle such as *Bos taurus* under stressful tropical environments (24). These are some examples of unique adaptive characteristics of the indigenous breeds, which evolved in stressful tropical environments enabling them to survive adverse environments. Therefore, it was hypothesized that indigenous pigs (Niang Megha) have better thermoregulatory mechanisms under changing climatic conditions of this region than exotic or crossbred pigs.

The objective of this study was set out to investigate the physiological, behavioral, and serum biochemical changes in response to thermal stress challenges in indigenous, crossbred, and exotic breeds of pigs.

## Materials and methods

### Location of the study

The present study was carried out in a pig farm of ICAR Research Complex for NEH Region, Umiam, Meghalaya. The study site is located at 25° 41′ 21″ N latitude and 91° 55′ 25″ E longitude with an altitude of 1,010 m above the mean sea level which falls in humid subtropical high rainfall area and receives rain in the range from 2,239 mm to 2,953 mm annually. In this region, the hottest months are usually July and August, while the coldest months are December and January.

### Meteorological measurements

The meteorological data were collected from the Division of System Research and Engineering (DSRE) of the institute during the experimental months. The temperature humidity index (THI) was calculated for each season using the formula, THI = 0.8T+ (RHT-14.4)/100 + 46.4 (25). The thermoneutral zone for a pig is between 18 and 20°C (26). The THI value ≤ 75 means no stress, 75 to 78 stressful, and ≥78 extreme stress (27). The THI value of 79–83 is considered danger, and exceeding this causes severe stress and death (28).

### Experimental animals and experimental design

Two experiments were conducted separately for summer (July–August) and winter (December–January) to study the effect of heat stress (summer) and cold stress (winter) on three genotypes of pigs, namely, indigenous (Niang Megha), Hampshire, and crossbred (having 75% Hampshire and 25% Niang Megha inheritance). Niang Megha is a registered indigenous pig breed of Meghalaya in India (accession no.: INDIA_PIG_1300_NIANGMEGHA_09002), with a small body size at maturity, having dense and long hairs and known for being better adapted to its native climatic conditions. Crossbred pig used in the present study is a crossbred pig variety called “Lumsniang” developed in the ICAR Research Complex for the Northeastern Hill Region, Meghalaya, for better adaptability and performance in the hill ecosystem of the Eastern Himalaya region. Six grower pigs (5–6 months) from each genetic group were selected for each experiment. The experimental animals were maintained in the pen system of housing and fed with balanced concentrate mesh feed two times daily and drinking water was provided *ad libitum* throughout the period. The experimental pigs were maintained under the standard and uniform managemental conditions. The animals were dewormed on a routine basis and regularly vaccinated. The welfare of the pigs was protected, and the study was approved by the Institutional Animal Ethics Committee (IAEC).

### Physiological responses to summer and winter

Physiological parameters including rectal temperature (T_R_), skin surface temperature (T_SS_), heart rate (HR), and respiration rate (RR) were recorded at 0,900 and 1,600 h weekly once for 2 months for each season, and the mean values were considered. Rectal temperature was recorded by inserting a clinical digital thermometer into the rectum of the pigs with proper restraining to avoid stress. The measurement for skin surface temperature was taken at the lumbar region of the pig using an infrared thermal imager Testo 875i (Testo India Pvt. Ltd., Pune, India). The emissivity of 0.95 was taken as the standard emissivity of the pig body surface in the study. Heart rate was determined by counting the number of heartbeats per minute using a stethoscope. Respiration rate was taken when the pig was at the resting phase by visual observation of flank movement to detect breaths per minute. All the parameters were taken by two trained personnel for all the experimental animals to avoid variation between individuals.

### Behavioral response to summer and winter

Behavioral mechanisms associated with heat and cold stress were observed in summer and winter, respectively. The pigs were monitored for different lying positions, such as huddling, shivering, standing, wallowing, and physical activity, by using CCTV video cameras. A total of six CCTV video cameras were used for this study. The thermoregulatory behavior patterns of each genetic group were recorded for 60 days in both the seasons. The duration (min) and frequency (%) for each activity during 24 h (from 6.00 a.m. until 6.00 a.m. of the next day) for 8 days were taken weekly once (for 8 weeks) in each season from pre-recorded videos for each animal.

### Serum biochemical changes in response to summer and winter

Blood samples were collected from all the experimental animals separately at 15-day interval during two experimental seasons. Five milliliters of blood samples was aseptically collected before feeding in the morning by venipuncture of the anterior vena cava using sterilized plastic disposable syringes from each animal. The collected samples were immediately transferred to a sterile serum separator vacutainer tube (Becton Dickinson, Franklin, USA), allowed to clot, and then centrifuged at 3,000 rpm for 15 min at room temperature. During blood collection, pigs were handled very carefully to avoid handling stress, and the experimental animals were restrained in the ventro-dorsal position. The collected sera samples were stored at −20°C until further analysis. To assess the metabolic and serum biochemical changes in grower pigs in response to heat stress (summer) and cold stress (winter), commercially available kits were used to analyze the following parameters: alkaline phosphatase (ALP), insulin, thyroxine (T4), superoxide dismutase (SOD), acid phosphatase, alanine transaminase (ALT), and cortisol. All the parameters were estimated as per the manufacturer's instruction.

### Statistical analysis

The data on physiological, behavioral, and serum biochemical parameters in different genetic groups and between the seasons were analyzed by comparing the means through multivariate analysis of variance (ANOVA). The data on behavioral activities were first transformed using log 10 to normalize it before analysis. The mean differences of different genetic groups for different parameters and between the seasons were tested for statistical significance (at a 5% level of significance) by Duncan's multiple range test (DMRT) using SPSS version 22.0 statistical software.

## Results

### Meteorological measurements

The maximum and minimum temperature, relative humidity, and temperature humidity index for the study period are presented in Table 1. The present study recorded a maximum THI of 93–96 and a minimum THI of 73–79 during the summer months. Similarly, a maximum THI of 83–87 and a minimum THI of 53–63 were recorded during winter. The highest temperature, relative humidity, and THI were recorded in the months of June (temp. 29.3°C, RH 86%, THI 95.89) and July (temp. 29.70°C, RH 88.90 %, THI 96.47) during the study period. The lowest temperature, relative humidity, and THI were recorded in January (temp. 5.50°C, RH 44.6%, THI 53.11) and February (temp. 8°C, RH 40.8%, THI 55.92).

**Table 1:** Maximum and minimum temperature, relative humidity, and THI recorded during experimental period.

**Season**	**Months**	**Temperature (** **°** **C)**		**R.H (%)**		**THI**	
		**Max**	**Min**	**Max**	**Min**	**Max**	**Min**
Summer	July	29.70	21.40	88.90	76.00	96.42	79.64
	August	28.00	20.90	90.50	77.30	94.00	79.13
Winter	December	22.90	8.60	86.40	54.70	84.36	57.84
	January	22.10	5.50	85.90	44.60	82.92	53.11

R.H, relative humidity; THI, temperature humidity index.

### Physiological measurements

Physiological measurements of different genetic groups during summer and winter are presented in Table 2. In the summer season, indigenous pigs had significantly lower (*p* < 0.05) T_R_ with no significant difference between Hampshire and crossbreed pigs. Skin surface temperature was significantly higher (*p* < 0.05) in Hampshire with no significant difference between indigenous and crossbred pigs. There were significant differences in RR and HR among the genetic groups. Indigenous pigs had significantly lower (*p* < 0.05) RR and HR followed by crossbred pigs and the highest was recorded in Hampshire.

**Table 2:** Means ± SE for physiological parameters of indigenous, Hampshire, and crossbred pigs during the study period.

**Physiological parameters**	**Seasons**	**Indigenous (*n* = 6)**	**Hampshire (*n* = 6)**	**Crossbred (*n* = 6)**
Rectal temperature (°C)	Summer	38.32 ± 0.06^a^	39.39 ± 0.02^b^	39.10 ± 0.05^b^
	Winter	38.04 ± 0.07^a^	38.59 ± 0.1^a^	38.49 ± 0.06^a^
Skin surface temperature (°C)	Summer	28.49 ± 0.03^a^	30.51 ± 0.3^b^	28.35 ± 0.03^a^
	Winter	21.66 ± 0.04^a^	21.58 ± 0.5^a^	21.64 ± 0.06^a^
Respiratory rate (breaths/min)	Summer	22.42 ± 0.13^a^	34.42 ± 0.06^b^	27.67 ± 0.11^c^
	Winter	17.17 ± 0.9^a^	20.17 ± 0.09^b^	17.83 ± 0.17^a^
Heart rate (bpm)	Summer	62.75 ± 0.17^a^	76.58 ± 0.15^b^	64.58 ± 0.18^c^
	Winter	55.42 ± 0.08^a^	63.25 ± 0.08^b^	52.75 ± 0.12^c^

Values are the average of measurements taken at different times (0,900 and 1,600 h for 4 days) of the day during each season. ^a − c^Values in the same row with different superscripts differ significantly (p < 0.05).

In the winter season, there were no significant differences in T_R_ and T_SS_ among the genetic groups. Although RR was significantly higher (*p* < 0.05) in Hampshire, but had no significant (*p* < 0.05) difference between indigenous and crossbred pigs. Heart rate had a significant (*p* < 0.05) difference among the genetic groups. Hampshire had significantly higher (*p* < 0.05) HR followed by indigenous, and the lowest was recorded in crossbred pigs.

### Behavioral mechanisms response to summer and winter

Different behavioral mechanisms were observed among the genetic groups when exposed to heat and cold stresses in the experimental animals (Table 3). Hampshire showed significantly (*p* < 0.05) higher duration and frequency of lateral and half-lateral lying positions in both the seasons followed by crossbred and indigenous pigs, while the duration and frequency of sternal lying were significantly (*p* < 0.05) higher in indigenous pigs followed by crossbred and Hampshire pigs. Similarly, indigenous pigs showed higher duration and frequency of standing and physical activity in both the seasons. Wallowing behavior was observed only in the summer season, while huddling and shivering behavior were observed in the winter season in all the genetic groups. Wallowing and shivering thermogenesis behavior were observed significantly (*p* < 0.05) highest in Hampshire followed by crossbred pigs during heat and cold stresses, respectively, whereas huddling behavior was observed significantly (*p* < 0.05) highest in indigenous pigs followed by crossbred pigs during cold stress.

**Table 3:** Means ± SE for behavioral parameters of indigenous, Hampshire, and crossbred pigs in response to heat and cold stress observed under a CCTV video camera during the experimental period.

**Behavioral parameters**	**Season**	**Duration (minutes/day)**			**Frequency/day (%)**		
		**Indigenous (*n* = 6)**	**Hampshire (*n* = 6)**	**Crossbred (*n* = 6)**	**Indigenous (*n* = 6)**	**Hampshire (*n* = 6)**	**Crossbred (*n* = 6)**
Lateral lying	Summer	204.02 ± 1.99^a^	613.69 ± 1.58^b^	453.69 ± 0.67^c^	14.17 ± 0.14^a^	42.62 ± 0.11^b^	31.51 ± 0.05^c^
	Winter	284.96 ± 0.57^a^	396.69 ± 1.43^b^	353.54 ± 0.51^c^	19.79 ± 0.04^a^	27.55 ± 0.1^b^	24.55 ± 0.04^c^
Sternal lying	Summer	364.38 ± 2.08^a^	105.38 ± 1.52^b^	215.19 ± 1.24^c^	23.30 ± 0.14^a^	7.32 ± 0.11^b^	14.94 ± 0.09^c^
	Winter	152.54 ± 0.57^a^	101.42 ± 1.45^b^	147.06 ± 0.85^a^	10.59 ± 0.04^a^	7.04 ± 0.1^b^	10.21 ± 0.06^a^
Half lateral lying	Summer	81.83 ± 1.82^a^	310.63 ± 0.00^b^	202.35 ± 1.35^c^	5.68 ± 0.13^a^	21.57 ± 0.27^b^	14.05 ± 0.09^c^
	Winter	77.38 ± 0.62^a^	119.88 ± 1.04^b^	111.42 ± 0.43^c^	5.37 ± 0.04^a^	8.32 ± 0.07^b^	7.74 ± 0.03^c^
Huddling	Summer	-	-	-	-	-	-
	Winter	404.52 ± 0.64^a^	380.73 ± 0.97^b^	401.42 ± 0.92^c^	28.09 ± 0.04^a^	26.44 ± 0.07^b^	27.88 ± 0.06^c^
Shivering	Summer	-	-	-	-	-	-
	Winter	79.69 ± 0.44^a^	146.27 ± 1.31^b^	120.79 ± 0.93^c^	5.53 ± 0.30^a^	10.16 ± 0.09^b^	14.19 ± 5.41^c^
Standing	Summer	411.35 ± 4.92^a^	92.19 ± 3.24^b^	205.23 ± 1.25^c^	28.57 ± 0.34^a^	6.40 ± 0.23^b^	14.25 ± 0.09^c^
	Winter	99.88 ± 2.02^a^	55.15 ± 1.76^b^	60.94 ± 1.30^c^	6.94 ± 0.14^a^	3.83 ± 0.12^b^	7.43 ± 3.01^c^
Wallowing	Summer	105.79 ± 1.59^a^	180.44 ± 2.38^b^	140.25 ± 1.33^c^	7.35 ± 0.11^a^	12.53 ± 0.17^b^	9.74 ± 0.09^c^
	Winter	-	-	-	-	-	-
Physical activity	Summer	272.63 ± 1.64^a^	137.69 ± 1.02^b^	223.29 ± 0.38^c^	18.93 ± 0.11^a^	9.56 ± 0.07^b^	15.51 ± 0.03^c^
	Winter	341.04 ± 1.14^a^	239.88 ± 0.66^b^	244.83 ± 2.16^c^	23.68 ± 0.08^a^	16.66 ± 0.05^b^	17 ± 0.15^c^

^(a − c)^Values in the same row with different superscripts differ significantly (p < 0.05).

### Serum biochemical response to summer and winter

The serum biochemical responses to heat and cold stresses are presented in Table 4. The result revealed that during heat stress, the serum concentrations of alkaline phosphatase (ALP) and thyroxine (T_4_) were significantly (*p* < 0.05) higher in indigenous pigs followed by crossbred and Hampshire. Among different genetic groups, the serum insulin level was significantly (*p* < 0.05) higher in indigenous pigs; however, there was no significant difference between crossbred and Hampshire. The serum concentrations of alanine transaminase (ALT) differed significantly (*p* < 0.05) among different genetic groups. It was significantly (*p* < 0.05) higher in crossbred followed by Hampshire and indigenous pigs. The activity of superoxide dismutase (SOD) and acid phosphatase showed no significant difference among the genetic groups during heat stress. The cortisol level was significantly (*p* < 0.05) lowest in indigenous pigs followed by crossbred and highest in Hampshire during heat stress.

**Table 4:** Means ± SE of indigenous, Hampshire, and crossbred pigs for serum biochemical parameters during the study period.

**Parameters**	**Seasons**	**Indigenous (*n* = 6)**	**Hampshire (*n* = 6)**	**Crossbred (*n* = 6)**
Alkaline phosphatase (ng/ml)	Summer	8.41 ± 0.12^a^	3.56 ± 0.01^b^	5.41 ± 0.03^c^
	Winter	6.46 ± 0.03^a^	1.66 ± 0.05^b^	3.48 ± 0.06^c^
Alanine transaminase (U/l)	Summer	22.16 ± 0.03^a^	28.26 ± 0.04^b^	38.23 ± 0.13^c^
	Winter	13.72 ± 0.03^a^	17.88 ± 0.4^b^	19.47 ± 0.09^c^
Insulin (μIU/ml)	Summer	7.01 ± 0.11^a^	4.18 ± 0.1^b^	4.8 ± 0.04^b^
	Winter	5.48 ± 0.03^a^	12.48 ± 0.05^b^	7.42 ± 0.03^c^
T_4_ (ng/ml)	Summer	38.64 ± 0.27^a^	5.52 ± 0.06^b^	18.08 ± 0.04^c^
	Winter	45.64 ± 0.25^a^	11.64 ± 0.04^b^	26.64 ± 0.11^c^
SOD (U/l)	Summer	1.91 ± 0.05	1.73 ± 0.03	1.86 ± 0.06
	Winter	1.83 ± 0.60	1.54 ± 0.01	1.79 ± 0.03
Acid phosphatase (μmol/min/ml)	Summer	0.042 ± 0.00	0.12 ± 0.01	0.049 ± 0.00
	Winter	0.07 ± 0.05	0.09 ± 0.00	0.026 ± 0.01
Cortisol (ng/ml)	Summer	28.5 ± 0.15^a^	56.8 ± 0.07^b^	40.2 ± 0.02^c^
	Winter	27.64 ± 0.01^a^	47.8 ± 0.02^b^	28.3 ± 0.24^a^

^(a − c)^Values in the same row with different superscripts differ significantly (p < 0.05).

Similarly, during cold stress the serum concentrations of alkaline phosphatase (ALP) and thyroxine (T_4_) were significantly (*p* < 0.05) higher in indigenous pigs followed by crossbred and Hampshire. The serum insulin levels and ALT concentrations were significantly (*p* < 0.05) different among the genetic groups. The insulin levels were recorded highest in Hampshire followed by crossbred and indigenous pigs; however, the ALT concentrations were recorded highest in crossbred pigs followed by Hampshire and indigenous pigs. No significant difference in SOD and acid phosphatase was observed among the genetic groups. The cortisol level was significantly (*p* < 0.05) higher in Hampshire with no significant difference between indigenous and crossbred pigs.

## Discussion

It is important to know that pigs are susceptible to heat and cold stresses. The present study revealed that the microclimate conditions of the present study location cause heat stress and cold stress in pigs during the summer and winter seasons, respectively. The maximum and minimum THI values during the summer season were above the established normal THI values of 75 or less for pigs (27). The THI values observed in the current study were those that have previously been reported to cause extreme stress in pigs and may even be fatal (27, 28). The primary response of animals under thermal stress is an increase in respiration rate, rectal temperature, and heart rate (29). Rectal temperature is a delayed indicator of heat stress only responding when the temperature is over 27°C or the THI is >80 (30, 31). In the present study, Hampshire and crossbred pigs had higher T_R_ during heat stress than indigenous pigs. Given that an increase in T_R_ is an indication of heat stress, higher T_R_ in Hampshire and crossbred pigs may indicate that they are more susceptible to heat stress. This is confirmed by the previous study, where pigs exposed to heat stress had a higher rectal temperature (32). While lower TR in indigenous pigs suggest that indigenous pigs might have a better thermoregulatory mechanism to dissipate heat from the body. T_R_ for indigenous and exotic pigs under heat stress, however, did not differ according to some studies (23, 33). During heat stress, Hampshire pigs were found to have higher T_SS_ than indigenous and crossbred pigs, which may indicate that they are more susceptible to heat stress. Elevated T_SS_ with an increase in ambient temperature was reported in pigs (23). Furthermore, exposure of ruminants to high environmental temperature also increased skin temperature (34), documented in Nguni and Boran cattle breeds (35) and Osmanabadi goats (36). This higher T_SS_ might be directly attributable to the vasodilatation of the skin capillary bed, which would enhance blood now to the cutaneous blood vessels, allowing more efficient heat transfer to the surroundings and sensible heat loss (36). The fact that indigenous and crossbred pigs had lower T_SS_ may be related to their long hair and high hair density, which serve as insulation and reduce heat loss, as demonstrated by Silanikove (30). Stress triggers the hypothalamus, which enhances respiratory activity to speed up heat escape from the body through respiratory evaporation (37). In animals, there is a correlation between respiration rate and the surrounding temperature and microenvironments (28). Thus, when animals are exposed to high ambient temperatures, they have an increased rate of respiration and perspiration (31). However, it was well-documented that porcine sweat glands are non-functional (19). Respiration rate is the first sign of heat stress and can be affected by temperatures as low as 21.3°C (31) or a THI of 73 (30). In the present study, RR of indigenous and crossbred pigs was within the normal range, that is, 15–30 breaths per minute as reported by Silanikove (30); however, it was higher than the normal range during heat stress in Hampshire, suggesting the susceptibility to heat stress. Among the genetic groups, higher RR in Hampshire and crossbred pigs during heat stress in the study could be the mechanism to dissipate more heat from the body by evaporating to the surrounding. Our finding of lower respiratory rate of indigenous pigs when exposed to heat stress corroborates with the finding of Moyo (38) in indigenous Windsnyer pigs of South Africa, which might be due to better adaptability of indigenous pigs to the agroclimatic condition of the region. An increase in RR was also reported in pigs exposed to high heat load (23, 39) and various cattle breeds (40). Heart rate is a stress marker that can be changed in response to thermal stress. Hampshire had higher HR than indigenous and crossbred pigs during heat stress, and this could be an attempt to dissipate excess heat to its surroundings by increasing blood flow to its peripheral tissues. A similar observation was earlier observed and documented by Madzimure et al. (23) who reported higher HR in large white than in indigenous Windsnyer pig in South Africa. Similar findings were also reported in sheep breeds reared in the Indian semiarid regions (41) and in other farm animals (36).

In the winter season, the temperature reaches a minimum of 5.5°C in the study location, which is below the lower limit of the thermoneutral zone (18–20°C) for a pig (26). Although pigs are susceptible to cold stress, there was not much variation in the physiological parameters during cold stress among the genetic groups. Pigs have a thick subcutaneous adipose tissue layer, which is attributed to maintain their normal physiological parameters during cold stress. Previous research has shown that when pigs are exposed to low temperature, they may increase heat production through muscular shivering thermogenesis (42), conserve heat through changing posture to reduce the body surface exposed to cold (43), build nests, huddling together, and select favorable microhabitats (42, 44). Similarly, on exposure to cold temperature, increased physical activity, huddling, and shivering were observed in the present study. Increased physical activity and huddling were observed highest in indigenous pigs followed by crossbred and Hampshire, while shivering was observed highest in Hampshire followed by crossbred pigs. Similarly, huddling together in cold temperature was observed in indigenous pigs (Windsnyer and Kolbroek) of South Africa (38). Increased physical activity was reported in large white (38) and crossbred (large white x Pietrain) pigs (45), which is contrary to the present study observation where it is highest in indigenous pigs.

Less time in standing and sitting and more time in lying postures in comparison with control during the cooling phase after the pigs are exposed to high temperature are reported in cooled sow (46). Similarly, more time in lying postures was observed during heat stress than cold stress and the highest lying behavior was observed in Hampshire followed by crossbred in the entire present study. Heat-stressed sows have been observed to show reduced standing posture (47) and consistently increase lying postures, especially lying laterally (48). Similarly, less standing time and more laterally lying posture were observed during heat stress, and the highest was observed in Hampshire followed by crossbred in the present study. Lying laterally appears to be a strategical posture when pigs suffer from heat stress, which is due to the skin surface in contact with the floor, which is greater than any other posture, enhancing heat loss through conduction (48). Reduced overall activity is one of the behavioral strategies used to help animals cope with heat stress. Physical activity was significantly reduced in heat stress in all the genotypes. Among the genetic groups, physical activity was observed highest in indigenous followed by crossbred than in Hampshire in both the seasons. Wallowing and standing behavior is one of the adaptive behavioral mechanisms to dissipate heat and reduce heat stress. When the heat load increased, animals spent more time standing to maximize heat dissipation by increasing the surface area of the skin exposed to air or wind flow (49). Standing behavior was observed highest in indigenous pigs, whereas wallowing behavior was highest in Hampshire. Pigs wallow for a variety of reasons, including cooling and protection from solar radiation and preventing attacks from insects (50). Wallowing allows pigs to lose heat more effectively during hot weather than sweating as well as during cold conditions; mud acts as insulation, allowing them to keep their body warm (14). However, wallowing behavior was not observed in all groups during the cold season in the present study as they are kept in an intensive system of rearing. Similar to previous findings, in this present study as well-different behavioral responses to conserve heat or dissipate heat during the cold and hot seasons were observed. In this study, indigenous pigs exhibited increased physical activity to generate body heat and huddled together in an attempt to conserve heat. On the contrary, Hampshire pigs attempt to increase heat production by shivering thermogenesis. Similarly, indigenous pigs showed the increased standing time to dissipate heat, while Hampshire and crossbred pigs presented a higher time of wallowing and reduced physical activity to reduce heat production. The fact that Hampshire and crossbred pigs spent more time in lying and wallowing could indicate that they were attempting to cool down to a comfortable temperature through evaporative cooling. However, physical activity was significantly higher and wallowing behavior was significantly lower in indigenous pigs followed by crossbred pigs. These behavioral responses indicate better homeostasis and adaptability of indigenous and crossbred pigs than Hampshire.

This study demonstrates that most of the metabolic and serum biochemical activities were influenced by thermal stress. Some metabolic enzymes increase their activity when exposed to high ambient temperatures. Among other enzymes, alanine aminotransferase (ALT) is one of the important metabolic enzymes that increased its activity when exposed to stress as frequently reported by several researchers. The increased activity of these enzymes was reported in goats (51), sheep (52), and pig (53, 54) during exposure to heat stress. A similar finding was observed in the present study. According to Banerjee et al. (55), an increase in the activity of this enzyme is related to the higher adaptive capability of the animals to cope with heat stress. It was also reported that ALT activity increases during stress (56), such as increased after severe exercise in humans (57) and restraint in rats (58). The enzyme activity of ALT was observed highest in Hampshire followed by crossbred pigs, suggesting that Hampshire pigs are more susceptible to stress than indigenous and crossbred pigs of this region. Acid phosphatase (ACP) and alkaline phosphatase (ALP) are two key enzymes involved in animal metabolism. The levels of these enzymes are generally low in heat-stressed animals, which could be attributed to a metabolic shift in the animals (51). Several investigations have reported a decrease in ALP activity in heat-stressed animals (53, 59), including pigs (60), which is contradictory to our present finding. Why ALP activity is higher during summer than winter in the present study is not known. It is thought that reduced ALP activity is attributed to reduce the functioning of the liver during heat stress exposure (61). The present observation revealed that the activity of serum ALP was highest in indigenous followed by crossbred pigs and lowest in Hampshire during the study periods. However, acid phosphatase did not differ significantly among genotypes in the present study. The above observations could suggest that indigenous pigs and crossbred are more resilient and have better adaptability to different climatic conditions than Hampshire.

The blood insulin concentration directly reflects the energy status of the animal to sustain production under extreme environmental conditions (62). An increase in basal and stimulated circulating insulin has been reported in a variety of species (63), including pigs (64), during heat stress. Ironically, Sejian et al. (17) reported a significant reduction in the insulin level after thermal exposure in goats. Similarly, we observed a higher level of insulin in indigenous pigs, which is inconsistent with the earlier findings (63, 64) and lower level of insulin in Hampshire and crossbred pigs (17) during heat stress. This difference in the level of insulin could be due to their nutritional status. A reduction in the level of insulin in Hampshire and crossbred pigs could be due to heat stress and resulted in reduced feed intake, as reported by Sejian et al. (65), and there was a significant reduction in the insulin level when thermal stress was combined with restricted feeding. Our findings also showed that exposure to a cold environment significantly reduced the level of insulin in indigenous pigs, but significantly increased in Hampshire and crossbred pigs. In rats, cold exposure decreased insulin secretion (66), stimulating the insulin signaling pathways in the brown adipose tissues for utilizing energy for thermogenesis (67). However, the increased level of insulin in Hampshire and crossbred pigs when exposed to a cold environment could be due to increased feed intake and elevated glucose concentration in the body, which stimulates the release of insulin.

The thyroid gland is extremely sensitive to temperature changes in the environment (68). Proper thyroid gland function and thyroid hormone activity are thought to be essential for domestic animals to maintain productive performance (69). Increased secretion of thyroid hormone increases body metabolism and hence heat production (28). Various researchers have reported a decrease in thyroid hormone (T_4_ and T_3_) blood concentrations in diverse species when exposed to heat stress (70, 71) including pigs (28). The serum concentrations of T_4_ and T_3_ were higher in cold stress than in heat stress conditions (52). The previous findings support the finding of the present study in that the serum concentration of T_4_ was shown to be significantly lower during heat stress. Reduced concentrations of circulating T_3_ and T_4_ have been reported in heifers (72), sheep (73), and goat (69), suggestive of an attempt to reduce metabolic rate and thus metabolic heat production. Overall, during the study periods, indigenous pigs demonstrated a significantly higher T_4_ serum concentration than either of the other genotypes. The lower level of T_4_ in Hampshire and crossbred pigs might indicate that they are more prone to cold and heat stresses than that in indigenous pigs. When an animal is exposed to heat, reduced food intake and metabolism slow down, which leads to negative energy balance and results in hypofunction of the thyroid gland (74). Consequently, Sejian et al. (17) reported a decrease in the level of thyroid hormones (T_3_ and T_4_) in stressed animals. In the present study, higher thyroid hormone (T_4_) secretion may be indicative of the superior adaptive capability of indigenous pigs to the different climatic conditions of this region.

Antioxidants, such as SOD and glutathione peroxidase, can efficiently eliminate reactive oxygen species (ROS) from the intracellular environment by detoxifying them (75). During hot and cold climate, there is an increase in the rate of reactive oxygen species (ROS) production leading to the activation of antioxidant enzymes to scavenge the ROS (76). Some researchers have also observed higher activity of SOD as a marker of oxidative stress in various species during hot ambient temperature (77). However, lower activity of SOD during heat stress pig was documented by Yang et al. (78). Kataria and Kataria (76) reported significantly higher activity of SOD during hot and cold environments as compared to moderate environment. However, in the present study, the enzymatic activity of SOD did not show a significant alteration among the genotypes during thermal stress.

The detection of cortisol is one of the most widely used methods to assess stress in animals. It has proven to be a major stress hormone and a reliable indicator of stress (79). An increase in circulating cortisol concentrations caused by the activation of the hypothalamic–pituitary–adrenal axis is one of the most frequent and general responses of an animal to stressful situations (30). An increase in serum cortisol levels is related to the increase in adrenocortical activity, a characteristic linked to the activation of the autonomic nervous system by stress (80). Therefore, higher serum cortisol concentrations in Hampshire during the study periods suggest that they are more vulnerable to heat and cold stresses. It was also observed that there was a significant increase in serum cortisol concentrations during summer in crossbred pigs, suggestive of heat stress. Similarly, higher cortisol level in heat-stressed pig was reported from arid tracts in Rajasthan, India (81). However, indigenous pigs of this region maintained the serum cortisol concentrations during thermal stress. The findings indicate that the climatic conditions of this region are stressful to Hampshire and crossbred pigs than indigenous pigs of this region.

## Conclusion

The results suggest that indigenous pigs had better adaptability to heat and cold stresses than Hampshire and crossbred pigs. The superior tolerance to thermal stress of indigenous pigs was associated with an ability to maintain their physiological and behavioral activities. Behavioral activities to heat loss or conserve heat such as shivering and wallowing were lower in indigenous pigs, but higher physical activity during thermal stress. The better adaptability is also related to higher metabolic activity, which is shown by higher T_4_ activity and lower serum cortisol concentrations. Also, the better adaptability of indigenous pigs might be due to their unique anatomical features such as small body size, short legs, and long and dense bristles. Further detailed studies need to be carried out for a better understanding of the thermoregulatory mechanism of indigenous pigs and identifying the gene responsible for the resilient traits and developed adaptation signature that will be useful for the mainstreaming in the breeding program under changing climatic conditions.

## Data availability statement

The raw data supporting the conclusions of this article will be made available by the authors, without undue reservation.

## Ethics statement

The animal study was reviewed and approved by Institutional Animal Ethics Committee (IAEC), ICAR Research Complex for NEH Region, Umiam, Meghalaya, India.

## Author contributions

Conception, design of study, and interpretation were done by KG. Data collection and analysis were performed by CG and NSS. The manuscript was drafted by CG, NSS, and KG. Critical revision of the manuscript was done by NMS. All authors contributed to the manuscript revision and have read and approved the final manuscript.
